# PK/PD-guided ceftazidime–avibactam dosing for multidrug-resistant *Pseudomonas aeruginosa* infection in a preterm infant with chylothorax and ascites: a case report

**DOI:** 10.3389/fphar.2026.1849617

**Published:** 2026-05-28

**Authors:** Wenhao Yuan, Xiaotan Zhao, Xiaoyan Liu, Xuwei Tao, Lingkong Zeng

**Affiliations:** Department of Neonatology, Wuhan Children’s Hospital (Wuhan Maternal and Child Healthcare Hospital), Tongji Medical College, Huazhong University of Science and Technology, Wuhan, China

**Keywords:** ceftazidime-avibactam, chylothorax, individualized medication, multidrug resistance, pharmacokinetics/pharmacodynamics, preterm infant, *Pseudomonas aeruginosa*

## Abstract

**Background:**

Multidrug-resistant *Pseudomonas aeruginosa* (MDR-PA) infection in preterm infants is associated with high mortality, and treatment options are limited. Ceftazidime-avibactam (CAZ-AVI) is a novel β-lactam/β-lactamase inhibitor combination, but data on its pharmacokinetic/pharmacodynamic (PK/PD)-guided use in preterm infants are scarce.

**Methods:**

We report a 33-week preterm infant with congenital chylothorax/ascites complicated by MDR-PA infection. Antimicrobial susceptibility testing revealed resistance to meropenem (MIC > 16 μg/mL) and ceftazidime (MIC > 32 μg/mL) but sensitivity to CAZ-AVI (MIC = 4 μg/mL). A PK/PD-guided regimen of CAZ-AVI 40–50 mg/kg (based on the ceftazidime component, every 8 h, prolonged infusion ≥2 h) was administered to achieve a %fT > MIC target of ≥ 70%, considering the preterm infant’s immature renal function and increased volume of distribution.

**Results:**

After initiation of CAZ-AVI, high-sensitivity C-reactive protein (hs-CRP) decreased from 30.00 mg/L to 7.21 mg/L within 5 days and normalized by day 7. Thoracic and abdominal drainage volumes declined significantly. The infant was successfully weaned from mechanical ventilation and discharged with clinical recovery. No drug-related adverse events were observed.

**Conclusion:**

PK/PD-based individualized CAZ-AVI therapy achieved a favorable clinical outcome in this preterm infant with MDR-PA infection. A dosing strategy targeting %fT> MIC ≥ 70% appears feasible and safe in neonates. This case provides quantitative PK/PD support for CAZ-AVI dosing in preterm infants, a population with limited existing evidence.

## 1 Introduction

Chylothorax is a relatively rare but critical condition in the neonatal period, with an incidence of approximately 1/24,000 to 1/1,000 (DeWitt and Michalczyk, 2019). Preterm infants are more prone to chylothorax due to immature development of the lymphatic system. Persistent loss of large volumes of chylous fluid can lead to hypoalbuminemia and marked reductions in immunoglobulins and lymphocytes, thereby severely compromising immune function and increasing the risk of secondary infection (Resch et al., 2022; Bellini et al., 2022). Moreover, invasive procedures such as prolonged mechanical ventilation, central venous catheterization, and indwelling drainage tubes further increase the risk of nosocomial infection.

*Pseudomonas aeruginosa* (PA) is one of the most common opportunistic pathogens in neonatal intensive care units, exhibiting intrinsic resistance and a propensity for acquired resistance (Horcajada et al., 2019). In recent years, the detection rate of multidrug-resistant *P. aeruginosa* (MDR-PA) in neonatal intensive care units has been rising. Carbapenems were previously considered the “last line of defense” against MDR-PA infections; however, the emergence of carbapenem-resistant *P. aeruginosa* poses a serious clinical challenge, and recent international guidelines recommend newer β-lactam/β-lactamase inhibitor combinations such as ceftazidime–avibactam for selected resistant gram-negative infections (Tamma et al., 2024).

Ceftazidime-avibactam (CAZ-AVI) is a novel β-lactam/β-lactamase inhibitor combination. Avibactam is a diazabicyclooctane compound that inhibits various β-lactamases, including KPC and AmpC, thereby restoring the antibacterial activity of ceftazidime against enzyme-producing resistant bacteria (Sophonsri et al., 2024; Shirley, 2018). Currently, CAZ-AVI is approved for adults with complicated intra-abdominal infections, hospital-acquired pneumonia, and carbapenem-resistant *Enterobacteriaceae* infections, but clinical evidence for its use in neonates, especially preterm infants, is extremely limited (Ftergioti et al., 2024; Pu et al., 2023).

Here, we report a case of a 33-week preterm infant with congenital chylothorax complicated by MDR-PA infection successfully treated with CAZ-AVI. We further discuss PK/PD-based dosing strategies to provide evidence supporting individualized antimicrobial therapy in this vulnerable population.

## 2 Case presentation

### 2.1 General information

An 18-day-old female preterm infant was transferred to the Department of Neonatology, Wuhan Children’s Hospital, on 7 February 2026, due to persistent thoracic and abdominal effusions since birth. She was the third child of her mother’s second pregnancy and was delivered by cesarean section at 33 weeks of gestation because of threatened preterm labor, massive bilateral fetal pleural effusions, and ascites.

The birth weight was 2.3 kg, and Apgar scores were 6, 8, and 10 at 1, 5, and 10 min, respectively. Immediately after birth, the infant developed severe respiratory distress requiring endotracheal intubation and mechanical ventilation. Right-sided thoracentesis with closed drainage was performed.

During hospitalization at the referring center, the infant received invasive mechanical ventilation, continuous thoracic and abdominal drainage, and empirical antimicrobial therapy, including cefoperazone-sulbactam, teicoplanin, and meropenem. Additional supportive treatments included blood product transfusion, intravenous immunoglobulin, oral levothyroxine, and subcutaneous octreotide. However, the effusions persisted without significant improvement, and the patient was subsequently transferred for further management.

This study was approved by the Ethics Committee of Wuhan Children’s Hospital (approval No. 2026R046 E01), and written informed consent was obtained from the patient’s legal guardians.

### 2.2 Admission findings

On admission, the infant’s vital signs were as follows: temperature 36.5 °C, heart rate 150 beats/min, respiratory rate 50 breaths/min (under mechanical ventilation), and blood pressure 60/40 mmHg. Body weight was 2.60 kg.

Physical examination revealed a preterm infant in a sedated state with poor responsiveness and mild jaundice. Breath sounds were coarse bilaterally, accompanied by moist rales. The abdomen was mildly distended but soft on palpation, with decreased bowel sounds. Pitting edema was observed in both lower extremities. Neurological examination showed generalized hypotonia, with diminished primitive reflexes, including sucking, rooting, grasp, and Moro reflexes.

Based on clinical findings, the initial diagnoses were as follows: (1) bilateral chylothorax; (2) ascites; (3) neonatal pneumonia; (4) neonatal respiratory failure; (5) birth asphyxia; (6) preterm infant (33 weeks of gestation) with low birth weight; and (7) neonatal hypothyroidism.

### 2.3 Auxiliary examinations

Laboratory investigations performed on 7 February 2026, showed a white blood cell count of 7.03 × 10^9^/L, hemoglobin of 155 g/L, and platelet count of 168 × 10^9^/L. High-sensitivity C-reactive protein (hs-CRP) was 1.91 mg/L, and procalcitonin (PCT) was elevated at 0.350 ng/mL. N-terminal pro–B-type natriuretic peptide (NT-proBNP) was significantly increased to 3267.00 pg/mL.

Liver function tests revealed elevated total bilirubin (234.3 μmol/L), direct bilirubin (25.3 μmol/L), and total bile acids (50.2 μmol/L), while alanine aminotransferase (2 U/L) and aspartate aminotransferase (12 U/L) were relatively low.

Coagulation analysis showed prolonged prothrombin time (16.4 s) and activated partial thromboplastin time (98.6 s, critical value), with decreased fibrinogen (1.06 g/L).

Imaging studies demonstrated bilateral subependymal cysts on cranial ultrasound. Thoracic ultrasound revealed bilateral pleural effusions (anteroposterior diameter: left 0.8 cm, right 0.5 cm). Gastrointestinal ultrasound showed significant ascites, with a depth of 2.7 cm in both upper quadrants and 3.2 cm in the lower abdomen. Echocardiography identified a patent foramen ovale with mild tricuspid regurgitation.

Pleural and ascitic fluid analysis showed a nucleated cell count of 7,366 × 10^6^/L, with a predominance of lymphocytes (90%–99%). Rivalta test and chylous test were initially negative. Biochemical analysis of pleural fluid showed total protein levels of 24.8–30.3 g/L and lactate dehydrogenase levels of 97–157 U/L.

Microbiological testing on 10 February 2026, including routine bacterial culture, yielded negative results.

## 3 Treatment course and outcome

**FIGURE 1:**
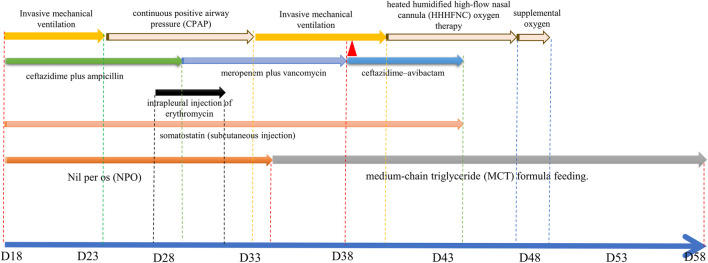
Timeline of Major Therapeutic Interventions During Hospitalization The horizontal axis represents the duration of hospitalization (days, D). The figure illustrates key interventions throughout the treatment course, including respiratory support (mechanical ventilation and non-invasive oxygen therapy), antimicrobial regimens (ampicillin plus ceftazidime, meropenem plus vancomycin, and ceftazidime–avibactam), erythromycin pleurodesis, somatostatin (octreotide) therapy, and initiation of medium-chain triglyceride (MCT) formula feeding prior to discharge. This figure provides a clear visualization of the temporal relationship between adjustments in anti-infective strategies and clinical interventions.

The main treatment measures and timeline of key events during hospitalization are shown in Figure 1.

### 3.1 Comprehensive supportive treatment

After admission to the neonatal intensive care unit, the infant received comprehensive supportive management. Respiratory support was provided via invasive mechanical ventilation using synchronized intermittent mandatory ventilation mode (FiO_2_ 30%–40%, respiratory rate 45–50 breaths/min, peak inspiratory pressure/positive end-expiratory pressure 21–23/5–6 cm H_2_O).

Continuous closed thoracic drainage and abdominal paracentesis drainage were maintained to relieve pleural and peritoneal effusions. Subcutaneous octreotide was administered to suppress lymphatic fluid production. In addition, intrapleural erythromycin (25–30 mg/kg every 24 h) was used for chemical pleurodesis.

Sedation and analgesia were achieved with continuous intravenous infusion of midazolam combined with fentanyl. Nutritional support consisted of total parenteral nutrition during the fasting period, followed by gradual transition to enteral feeding with a medium-chain triglyceride (MCT) formula via nasogastric tube.

Blood products, including red blood cells, plasma, albumin, and intravenous immunoglobulin, were administered as clinically indicated. Additional supportive measures included oral levothyroxine supplementation, phototherapy for hyperbilirubinemia, and correction of electrolyte imbalance and acid–base disturbances.

### 3.2 Anti-infective treatment course

#### 3.2.1 Phase 1: empirical anti-infection therapy (February 7–18, 2026)

Given the presence of multiple infection risk factors, including prolonged drainage, invasive ventilation, and immunocompromised status, empirical antimicrobial therapy was initiated with intravenous ampicillin (50 mg/kg every 8 h) combined with ceftazidime (40 mg/kg every 12 h). During this period, inflammatory markers and drainage volumes were closely monitored. Despite treatment, thoracic drainage remained persistently elevated (132–196 mL/day), with turbid, blood-tinged fluid, suggesting inadequate infection control.

#### 3.2.2 Phase 2: escalated anti-infection therapy (February 19–28, 2026)

Due to the lack of clinical improvement, antimicrobial therapy was escalated to meropenem (40 mg/kg every 8 h) combined with vancomycin (15 mg/kg every 8 h) on 19 February 2026, to broaden antimicrobial coverage. However, inflammatory markers continued to fluctuate, with hs-CRP ranging from 7.52 to 40.38 mg/L and reaching 30.00 mg/L on February 25. Thoracic drainage volume also increased to 210 mL/day, raising suspicion of infection with multidrug-resistant Gram-negative organisms.

#### 3.2.3 Phase 3: pathogen-confirmed targeted therapy (February 23–March 11, 2026)

On February 23, respiratory tract culture identified *P. aeruginosa* resistant to meropenem (MIC >16 μg/mL) and ceftazidime (MIC >32 μg/mL), but susceptible to ceftazidime-avibactam (MIC = 4 μg/mL). A specimen from the referring hospital also yielded KPC-producing *P. aeruginosa*, confirming MDR-PA infection.

A multidisciplinary team (MDT) consultation was conducted, involving specialists from pharmacy, imaging, interventional radiology, hematology, and immunology. Based on susceptibility results and the patient’s immature renal function, CAZ-AVI was recommended while avoiding aminoglycosides and polymyxins. After informed consent was obtained, CAZ-AVI therapy was initiated on 25 February 2026.

##### 3.2.3.1 CAZ-AVI regimen

Based on domestic and international neonatal dosing recommendations and the individualized regimen formulated by our hospital’s pharmacy department (Asfour et al., 2022; Bradley et al., 2025), The dosing regimen consisted of 40–50 mg/kg (based on the ceftazidime component) every 8 h, administered as an extended intravenous infusion over ≥2 h. Meropenem and vancomycin were discontinued on 28 February 2026.

To provide a structured overview of antimicrobial decision-making throughout hospitalization, the evolution of anti-infective regimens, underlying rationale, and corresponding clinical responses are summarized in Table 1. Notably, the transition to ceftazidime–avibactam was guided by antimicrobial susceptibility results and pharmacokinetic/pharmacodynamic (PK/PD) considerations, representing a shift from empirical and escalation therapy to targeted, individualized treatment.

**TABLE 1:** Anti-infective regimens, rationale for adjustment, and clinical responses during hospitalization.

Time period	Antimicrobial regimen	Dosage regimen	Rationale for adjustment	Clinical response
Feb 7–18, 2026	Ampicillin + ceftazidime	Ampicillin 50 mg/kg q8h; ceftazidime 40 mg/kg q12 h	Empirical therapy targeting common neonatal pathogens, including group B *Streptococcus* and gram-negative bacteria	Poor infection control; persistently high drainage volume
Feb 19–28, 2026	Meropenem + vancomycin	Meropenem 40 mg/kg q8h; vancomycin 15 mg/kg q8h	Escalation therapy to broaden coverage against ESBL-producing organisms and resistant gram-positive bacteria	Fluctuating hs-CRP levels; no significant improvement in drainage volume
Feb 25–Mar 11, 2026	Ceftazidime–avibactam	40–50 mg/kg q8h (based on ceftazidime component), prolonged infusion ≥2 h	Targeted therapy guided by susceptibility testing (MDR *Pseudomonas aeruginosa*, KPC-producing); PK/PD optimization aiming for %fT > MIC ≥ 70%	Rapid decline in hs-CRP; marked reduction in drainage volume; successful ventilator weaning

### 3.3 Clinical outcomes

#### 3.3.1 Inflammatory response

Prior to initiation of ceftazidime–avibactam (25 February 2026), the hs-CRP level was markedly elevated at 30.00 mg/L. After 5 days of therapy (2 March 2026), hs-CRP decreased to 7.21 mg/L, representing a 76% reduction. By day 7 of treatment (4 March 2026), hs-CRP further declined to 2.98 mg/L, returning to the normal range. Procalcitonin (PCT) also normalized during treatment. These findings indicate a rapid and sustained resolution of systemic inflammation.

#### 3.3.2 Drainage volume

**FIGURE 2:**
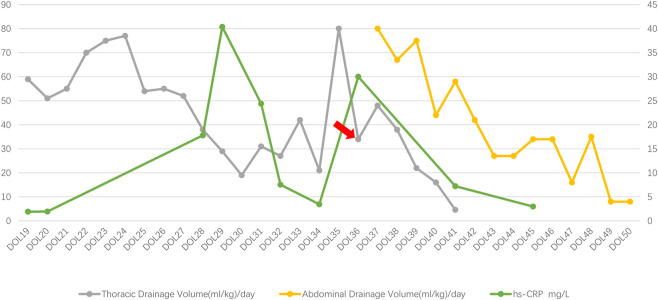
Trends in Inflammatory Markers and Drainage Volume During Treatment The horizontal axis represents the duration of hospitalization (days, d). The left vertical axis indicates high-sensitivity C-reactive protein (hs-CRP, mg/L), while the right vertical axis represents thoracoabdominal drainage volume (mL/d). The red arrow indicates the initiation of ceftazidime–avibactam (CAZ-AVI). Following CAZ-AVI therapy, hs-CRP levels decreased markedly, accompanied by a gradual reduction in thoracoabdominal drainage volume, suggesting effective control of infection.

Thoracic drainage decreased progressively from 210 mL/day prior to treatment (24 February 2026) to 12 mL/day at the time of chest tube removal (2 March 2026). Similarly, abdominal drainage decreased from 151 mL/day (2 March 2026) to 70 mL/day (4 March 2026), and further to 20 mL/day by 11 March 2026, allowing removal of the abdominal drain. Notably, chylous testing of pleural and ascitic fluid became positive during recovery, consistent with restoration of lymphatic flow. The dynamic trends of inflammatory markers and drainage volumes are presented in Figure 2.

#### 3.3.3 Respiratory recovery

The patient was successfully extubated on 2 March 2026, and transitioned to non-invasive positive pressure ventilation. Complete weaning from respiratory support was achieved on 7 March 2026, with stable spontaneous breathing thereafter.

#### 3.3.4 Imaging findings

**FIGURE 3:**
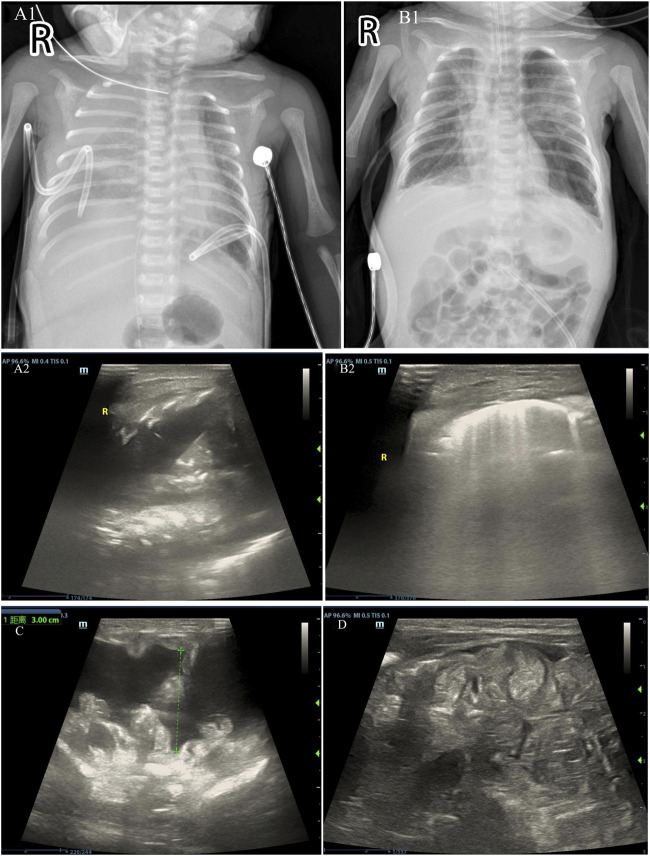
Comparison of Chest Imaging and Abdominal Ultrasound Findings Before and After Treatment. **(A1, A2)** Pre-treatment chest X-ray/ultrasound showing massive bilateral pleural effusion. **(B1, B2)** Post-treatment chest X-ray/ultrasound indicating near-complete resolution of pleural effusion. **(C)** Pre-treatment abdominal ultrasound showing massive ascites. **(D)** Post-treatment abdominal ultrasound demonstrating marked reduction of ascites. These findings indicate that chylothorax and chylous ascites were significantly improved following comprehensive therapy.

Follow-up bedside ultrasonography on 9 March 2026, demonstrated near-complete resolution of bilateral pleural effusion and significant absorption of pulmonary consolidation. Gastrointestinal ultrasonography on 15 March 2026, revealed only minimal residual ascites. Comparative imaging findings before and after treatment are shown in Figure 3.

#### 3.3.5 Clinical outcome at discharge

The infant was discharged on 19 March 2026, after a total hospitalization of 40 days. At discharge, the patient had stable vital signs, adequate spontaneous respiration, and was tolerating enteral feeding with medium-chain triglyceride formula (70 mL every 3 h). Final diagnoses included bilateral chylothorax with ascites, neonatal bronchopneumonia, *P. aeruginosa* enteritis, coagulation dysfunction, neonatal hypothyroidism, and patent foramen ovale.

#### 3.3.6 Safety assessment

Renal function, liver function, and auditory function were closely monitored throughout treatment. No drug-related adverse events, including nephrotoxicity, hepatotoxicity, or neurotoxicity, were observed.

## 4 Discussion

### 4.1 Anti-infective challenges in neonatal chylothora

Congenital chylothorax is a rare but clinically significant condition caused by abnormal lymphatic development, leading to the accumulation of chylous fluid in the pleural cavity (DeWitt and Michalczyk, 2019; Wang et al., 2022). In preterm infants, this condition is often more severe due to immature lymphatic and immune systems. Persistent chyle loss results in depletion of lymphocytes, immunoglobulins, and proteins, which substantially impairs immune function and predisposes to severe infections (Resch et al., 2022; Bellini et al., 2022).

In the present case, multiple risk factors for infection were identified, including prolonged invasive mechanical ventilation, indwelling drainage catheters, repeated transfusions, and ongoing immunosuppression secondary to chyle loss. These factors likely contributed synergistically to the development of multidrug-resistant *P. aeruginosa* infection (De Rose et al., 2025). This highlights the importance of implementing bundled infection prevention strategies in such high-risk neonates, including early removal of invasive devices, strict hand hygiene, and antimicrobial stewardship (Du et al., 2025).

### 4.2 Resistance mechanisms and interpretation microbiological interpretation

*Pseudomonas aeruginosa* is a major opportunistic pathogen in neonatal intensive care units, characterized by both intrinsic and acquired resistance mechanisms, which contribute to the rapid emergence of multidrug-resistant and extensively drug-resistant strains (Horcajada et al., 2019). These include β-lactamase production (e.g., AmpC, KPC), reduced outer membrane permeability (e.g., OprD loss), efflux pump overexpression, and biofilm formation (Infectious Diseases Group and Chinese Thoracic Society, 2022; Zhao et al., 2024).

In this case, the isolate demonstrated resistance to both meropenem and ceftazidime, and was confirmed to produce KPC carbapenemase, consistent with a multidrug-resistant phenotype. This resistance profile aligns with global surveillance trends, which indicate increasing carbapenem resistance rates in *P. aeruginosa* (World Health Organization, 2021; Zhang et al., 2023). Importantly, susceptibility testing revealed retained sensitivity to ceftazidime–avibactam, supporting its use as targeted therapy. This treatment strategy is also consistent with current IDSA guidance for antimicrobial-resistant gram-negative infections, which emphasizes susceptibility-guided use of novel β-lactam/β-lactamase inhibitor combinations (Tamma et al., 2024).

### 4.3 Pharmacological rationale for ceftazidime-avibactam

Ceftazidime–avibactam combines a third-generation cephalosporin with a novel non-β-lactam β-lactamase inhibitor. Avibactam effectively inhibits class A (including KPC) and class C β-lactamases, restoring ceftazidime activity against resistant organisms (Sophonsri et al., 2024; Shirley, 2018).

*In vitro* studies have demonstrated high susceptibility rates (85%–95%) of KPC-producing *P. aeruginosa* to ceftazidime–avibactam (Tumbarello et al., 2025). In this patient, the MIC of 4 μg/mL was well below the susceptibility breakpoint, suggesting a high likelihood of clinical efficacy. However, it is critical to emphasize that ceftazidime–avibactam lacks activity against metallo-β-lactamases, necessitating careful interpretation of resistance mechanisms prior to use.

### 4.4 PK/PD-guided dosing strategy in neonates

Ceftazidime–avibactam exhibits time-dependent antibacterial activity, with efficacy driven by the proportion of the dosing interval during which free drug concentrations exceed the MIC (%fT > MIC). A target of ≥50–70% is generally recommended for optimal efficacy (Han et al., 2025).

In this case, a more aggressive target (%fT>MIC ≥70%) was selected due to the severity of infection and limited therapeutic alternatives. Neonatal pharmacokinetics differ significantly from those of older populations, particularly in preterm infants, who exhibit increased volume of distribution, reduced protein binding, and immature renal clearance (Butranova et al., 2023). Existing literature consists mainly of case reports or small case series (Ftergioti et al., 2024; Pu et al., 2023; Asfour et al., 2022; Bradley et al., 2025; Pu et al., 2022; Zarras et al., 2023; Lockowitz et al., 2025).

These factors were carefully considered in dose selection. The regimen of 40–50 mg/kg every 8 h, administered as a prolonged infusion (≥2 h), was designed to maximize time above MIC while minimizing toxicity. The use of extended infusion is particularly important in cases where MIC values approach susceptibility breakpoints.

### 4.5 Quantitative PK/PD considerations

Using published neonatal pharmacokinetic parameters for ceftazidime, the selected regimen was estimated to achieve a %fT>MIC of approximately 75%–85% at an MIC of 4 μg/mL, exceeding the predefined target. The patient’s hypoalbuminemia likely further increased the free drug fraction, enhancing antibacterial activity.

The rapid clinical response—reflected by a 76% reduction in hs-CRP within 5 days—provides indirect validation of adequate PK/PD target attainment. Nonetheless, these estimates are based on population models, and the absence of therapeutic drug monitoring represents a limitation.

**FIGURE 4:**
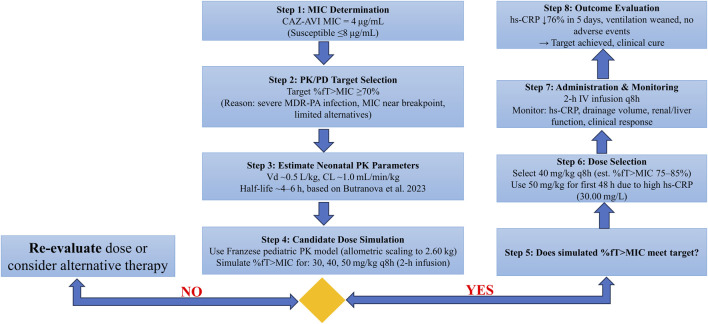
Decision flow diagram of the PK/PD-guided ceftazidime-avibactam dosing process. The diagram illustrates the eight sequential steps taken by the multidisciplinary team to individualize the CAZ-AVI regimen for this preterm infant with MDR-PA infection. Steps include: (1) MIC determination; (2) PK/PD target selection (%fT > MIC ≥ 70%); (3) estimation of neonatal PK parameters based on developmental pharmacology data; (4) simulation of %fT > MIC for candidate doses using the Franzese pediatric population PK model with allometric scaling; (5) decision check (target attainment?); (6) dose selection (40-50 mg/kg q8h, 2-h infusion); (7) administration and monitoring; (8) outcome evaluation. The rapid clinical improvement confirmed that the PK/PD target was achieved.

To further support the rationale of the selected dosing regimen, a structured PK/PD-based evaluation incorporating pathogen susceptibility, neonatal pharmacokinetic parameters, and estimated target attainment is presented in Table 2. To enable replication of our dosing decision, we describe the PK/PD-guided approach stepwise (summarized in Figure 4). This framework may facilitate implementation of PK/PD-guided antimicrobial dosing in similar NICU settings.

**TABLE 2:** PK/PD-based evaluation of ceftazidime–avibactam dosing regimen in this preterm infant.

Parameter	Estimated value	Source/Assumption	Clinical implication
Pathogen	*Pseudomonas aeruginosa* (MDR, KPC-producing)	Culture and susceptibility testing	Indicates limited treatment options; requires targeted therapy
MIC (CAZ-AVI)	4 μg/mL	Microbiology laboratory result	Near susceptibility breakpoint; requires optimized exposure
Dose	40–50 mg/kg q8h (ceftazidime component)	Based on neonatal case reports and institutional protocol	Within reported neonatal dosing range
Infusion duration	≥2 h (extended infusion)	PK/PD optimization strategy	Increases %fT>MIC
Volume of distribution (Vd)	∼0.4–0.6 L/kg	Published neonatal PK data	Larger Vd → lower peak concentration
Clearance (CL)	∼0.8–1.2 mL/min/kg	Neonatal PK literature	Reduced clearance → prolonged half-life
Half-life (t½)	∼4–6 h	Derived from PK studies	Supports q8h dosing interval
Protein binding	∼70–80% (ceftazidime)	Literature data	Hypoalbuminemia increases free fraction
Estimated %fT>MIC	∼75–85%	PK simulation based on above parameters	Achieves target ≥ 70%
PK/PD target	≥70% fT>MIC	Severe infection setting	More aggressive than standard target
Clinical response	Rapid CRP decline; drainage reduction; respiratory recovery	Observed outcome	Confirms adequacy of PK/PD target attainment

### 4.6 Role of therapeutic drug monitoring (TDM)

Therapeutic drug monitoring for ceftazidime–avibactam is increasingly recommended in critically ill patients, particularly in the presence of altered pharmacokinetics or infections with borderline MIC values. Although TDM was not performed in this case, the favorable clinical response suggests adequate drug exposure.

Future studies should integrate TDM into neonatal PK/PD research to establish exposure-response relationships and optimize individualized dosing strategies.

### 4.7 Importance of multidisciplinary management

Management of multidrug-resistant infections in neonates requires a multidisciplinary approach (Tong and Zhou, 2021). In this case, collaboration among neonatology, clinical pharmacy, microbiology, imaging, and immunology specialists was essential.

Pharmacists contributed to dose optimization based on PK/PD principles, while other specialists provided support in differential diagnosis and procedural management. This integrated approach ensured a rational, individualized, and safe therapeutic strategy.

### 4.8 Clinical implications and limitations

This case provides several important clinical insights:

1. Early and repeated pathogen identification is essential in high-risk neonates.
2. Ceftazidime–avibactam is a viable option for KPC-producing MDR *P. aeruginosa* infections when guided by susceptibility testing.
3. PK/PD-based dosing strategies are feasible in preterm infants and may improve outcomes.
4. Multidisciplinary collaboration is critical for optimizing complex anti-infective therapies.

However, this study has limitations, including its single-case design, lack of TDM data, and short follow-up duration. Additionally, genomic characterization of the pathogen was not performed. Larger prospective studies are needed to validate these findings.

## 5 Conclusion

PK/PD-guided ceftazidime–avibactam therapy achieved favorable clinical efficacy and short-term safety in a preterm infant with chylothorax complicated by multidrug-resistant *P. aeruginosa* infection. This case highlights that individualized dosing based on neonatal pharmacokinetic and pharmacodynamic principles is both feasible and clinically meaningful in critically ill preterm infants.

In particular, a dosing regimen of 40–50 mg/kg every 8 h administered as a prolonged infusion (≥2 h) was able to achieve a target of %fT>MIC ≥70%, which was associated with rapid resolution of inflammation, reduction in drainage volume, and successful respiratory recovery.

However, the use of ceftazidime-avibactam in neonates should strictly adhere to key principles: (1) administration should be guided by confirmed antimicrobial susceptibility; (2) dosing should be individualized according to developmental pharmacokinetics and renal function; (3) multidisciplinary collaboration is essential to optimize therapeutic decision-making; and (4) close monitoring of efficacy and safety is required throughout treatment.

Given the limited evidence base, further multicenter prospective studies incorporating therapeutic drug monitoring and population PK/PD modeling are warranted to validate optimal dosing strategies and to better define the efficacy and safety of ceftazidime–avibactam in neonatal populations.

## Data Availability

The original contributions presented in the study are included in the article/supplementary material, further inquiries can be directed to the corresponding author/s.
